# Rare Presentation of Acute Myeloid Leukemia With TP53 Mutation and Dermatologic Manifestations

**DOI:** 10.7759/cureus.37012

**Published:** 2023-04-01

**Authors:** Ronald Lott, Sara Stawitzky, Alexandra Stroia, Angela Awad, Anthony Kam, Maxim Bleicher

**Affiliations:** 1 Medicine, Lake Erie College of Osteopathic Medicine, Erie, USA; 2 Family Medicine, Arnot Ogden Medical Center, Elmira, USA

**Keywords:** acute myeloid leukemia (aml), mortality in leukemia, leukemoid reaction, leukemia cutis, tp53 mutations

## Abstract

Acute myeloid leukemia (AML) is a complex and aggressive malignancy that occurs due to genetic mutations and subsequent stem cell overproduction. We report a case of a patient with AML and a highly fatal, uncommon TP53 mutation who developed dermatologic manifestations. This report serves to highlight the importance of dermatologic findings in underlying leukemia and educate healthcare providers on the diagnosis and treatment of a rare TP53 mutation in AML.

## Introduction

Acute leukemias are responsible for approximately 10,000 cancer mortalities each year in the United States. Acute myeloid leukemia (AML), in particular, is diagnosed in 3.5 per 100,000 adults yearly, with an increased incidence in the elderly population [1]. AML is the result of a multi-step process involving genetic aberrations, which allow for the formation of preleukemic and leukemic stem cells that are capable of proliferating and causing the clinical condition of leukemia [2]. Early identification of various tumor suppressors and oncogenes can aid in determining the prognosis, classification, and treatment of many AML cases. Common recurrent mutations associated with AML pathogenesis have been found in FLT3, NPM1, KIT, CEBPA, TET2, DNMT3A, RUNX1, and IDH1. Risk stratification based on mutations continues to be described with a clear unfavorable prognosis associated with TP53 mutations [3].

Clinical presentations of AML often vary and are non-specific due to the heterogeneity of the disease. Symptoms associated with pancytopenias, such as fever, weakness, and bleeding, are most common, with fewer patients presenting with generalized pain, cutaneous findings, lymphadenopathy, or hepatosplenomegaly [4]. Cutaneous findings, in particular, can aid in diagnosis and indicate prognosis in patients with AML. These findings are highly variable and may represent non-specific findings such as erythema nodosum, cutaneous small vessel vasculitis, and acute neutrophilic dermatosis or specific cutaneous infiltration by malignant myeloproliferative cells, as seen in leukemia cutis. The presence of leukemia cutis is typically associated with decreased survival time and a poor prognosis. Cutaneous findings secondary to underlying immunosuppression and hematologic dysfunction, such as petechiae, pallor, bruising, and bacterial infections, are also often seen [5]. 

This case report details a unique patient with AML who presented with cutaneous skin lesions as well as a rare and highly unfavorable mutation of the TP53 tumor suppressor gene. The goal of this report is to convey the importance of considering underlying malignancies in patients with dermatological findings as well as to inform healthcare providers of the increased mortality associated with TP53 mutations in AML.

## Case presentation

A 73-year-old male with a past medical history of congestive heart failure, hypogonadism, and acute coronary syndrome initially presented to the emergency department of the Arnot Ogden Medical Center in Elmira, New York, with complaints of chest pain and vomiting. During workup for acute coronary syndrome, the patient was found to have a new onset normocytic anemia with a hemoglobin of 7.3, hematocrit of 21.5, mean corpuscular volume (MCV) of 91.6, and a negative fecal occult blood test. Iron studies demonstrated an iron of 222 uG/dL (low), an iron binding capacity of 232 uG/dL (low), and a ferritin of 938 NG/mL (high) suggesting an anemia of chronic disease. A follow-up manual differential demonstrated elevated bands of 10%, 2% myelocytes, 4% nucleated RBCs, and giant platelets, as well as anisocytosis, hypochromasia, microcytes, ovalocytes, schistocytes, acanthocytes, and elliptocytosis. Furthermore, the smear demonstrated scattered atypical immature mononuclear cells. The patient was also found to have an equivocal Symphony ELiA IgG Assay (Thermo Fisher Scientific Inc., Waltham, Massachusetts) for antinuclear antibodies. Additional laboratory studies during the patient's hospital stay demonstrated a normal total bilirubin of 0.7 mg/dL and a negative direct Coombs test suggesting no hemolysis. At this time, it was recommended that the patient follow up with hematology-oncology for a bone marrow biopsy and flow cytometry after receiving treatment for his non-ST elevation myocardial infarction.

Bone marrow aspiration with karyotyping and fluorescence in situ hybridization (FISH) analysis demonstrated findings indicative of AML, including significant dysplasia of all cell lineages and 32% blasts with a high nucleus-to-cytoplasmic ratio, immature chromatin, and 1-2 nucleoli within the aspirate. However, there was also an increased amount of atypical erythrocyte precursors preventing the medical team from ruling out pure erythroid leukemia. Additional somatic mutation analysis demonstrated TP53 mutations (p.Q104, 29% variant allele frequencies) and SRSF2 mutations (p.P95H, 3% variant allele frequencies). Additionally, a TP53 mutation of unknown significance was found in 48% of variant allele frequencies. TP53 mutations are only found in 4% of AML cases and tend to be associated with a higher mortality rate [6]. A chromosomal analysis of 20 cells revealed clonal aberrations in 18/20 cells. The analysis of these mutations is further depicted in Table 1.

**Table 1:** Mutations demonstrated in an analysis of 20 cells; 18/20 cells were found to have aberrations

Mutation type	Location
TP53 variant	Variant with known pathogenic mutation detected in 29% of variant allele frequencies, variant with unknown significance detected in 48% of variant allele frequencies
Deletions	5q, 7q
Derivative chromosomes	2p24, 13q12
Terminal deletion	Chromosome 3, band 3p25
Interstitial deletions	Long arm chromosome 5 with breakpoints at 5q12 and 5q33, long arm chromosome 11 with breakpoints at 11q13 and 11q23
One copy loss	Chromosome 7, chromosome 13

**Figure 1:**
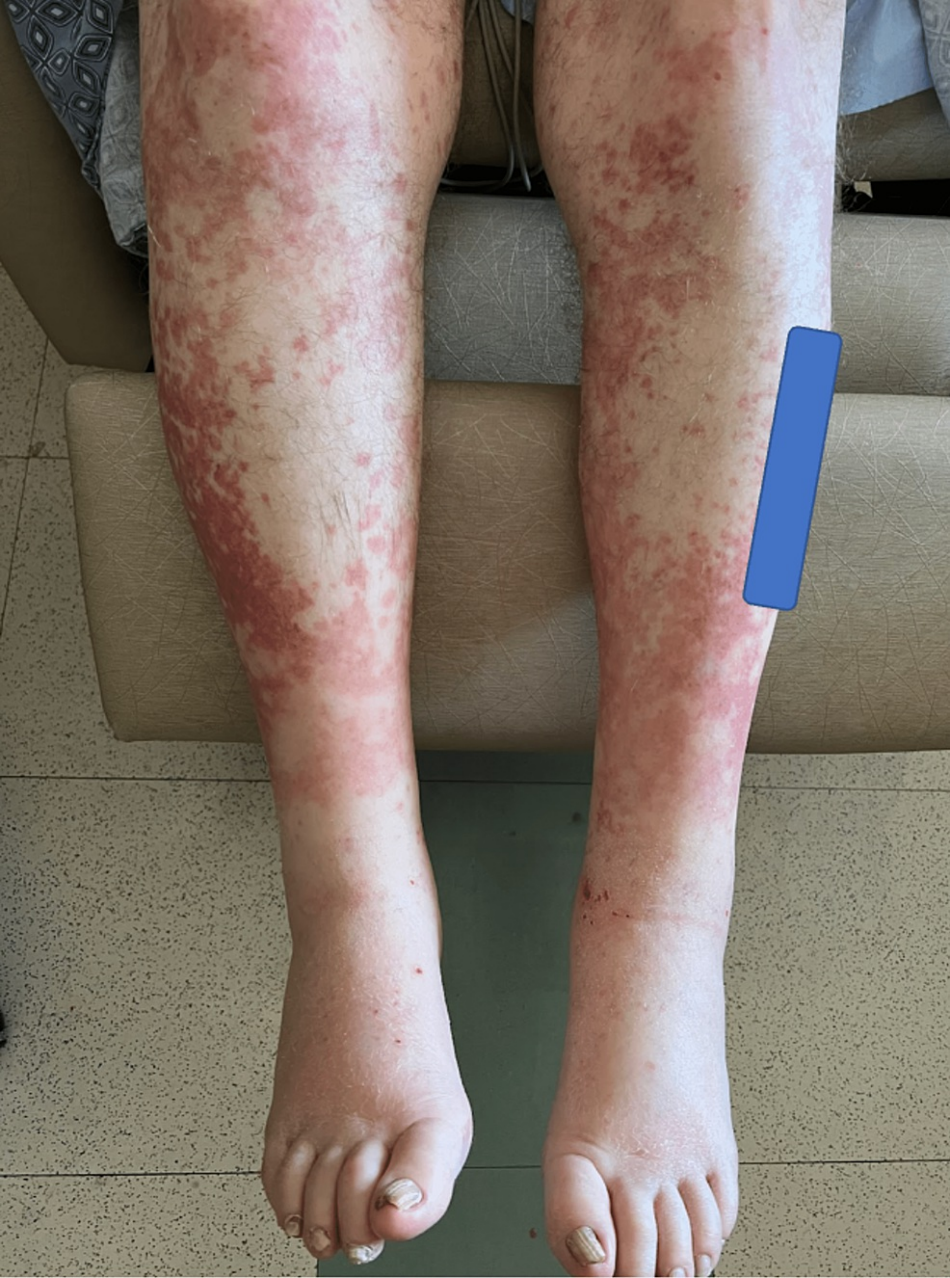
Generalized infiltrative papular, pruritic rash of the bilateral lower extremities which extends to the abdomen A blue image block was used to occlude patient identifying information on the left leg

Soon after diagnosis, the patient presented to the emergency department complaining of a worsening rash. On admission, a generalized infiltrative deep red, pruritic, maculopapular rash coalescing into erythematous patches was observed on the bilateral lower extremities and low abdomen (Figure 1). The patient stated that the rash initially began on his right ankle spreading to both of his lower extremities as well as the lower half of his abdomen. He reported that this rash was associated with a burning and itching pain, rating it as a 20/10 on the pain scale. Additionally, he admitted to some numbness and discoloration of his fingers bilaterally. At this time, the primary team was uncertain whether the rash was related to the patient's AML, a drug reaction, or a vasculitis such as Henoch-Schonlein purpura. Hematology-oncology was consulted and recommended initiation of diphenhydramine and prednisone for the rash. The patient was started on diphenhydramine 25 mg daily every six hours as needed for itching and burning, as well as prednisone 40 mg and topical triamcinolone for five days. On day three of the hospital course, the patient reported a moderate reduction in his itching. By day four, the rash had subsided, and the patient was discharged on day five of receiving these medications. 

Despite improvement, one month after discharge, the rash persisted, and the patient was referred to dermatology for a punch biopsy of the skin to rule out leukemia cutis. A punch biopsy of the right thigh demonstrated sections of dermal fibrosis with slightly ectatic superficial dermal vessels. Despite the clinical appearance of potential leukemia cutis, there was no significant evidence to support hematolymphoid processes in the skin. However, as this biopsy was taken while the rash was resolving and only a single site was sampled, leukemia cutis could still be considered.

In spite of an estimated prognosis of fewer than six months, the patient requested to proceed with treatment via azacitidine. After attempting treatment for three months, the patient was unable to tolerate chemotherapy and opted to enroll in hospice care. 

## Discussion

Our patient with AML was found to have a rare and often more fatal TP53 mutation. Overall, TP53 mutations are found in 5-15% of myeloid leukemias and are more common in older populations. In elderly patients, TP53 mutations are found in 25% of AML cases [7]. The role of TP53 in tumor suppression makes a loss of this gene detrimental to the health of the individual due to uncontrolled cellular proliferation. A 2012 study completed by Grossman et al. found that in a population of 80 patients with AML and TP53 mutations, 0% survived greater than three years [8]. This study also analyzed molecular mutations of PML-RARA, RUNX1-RUNX1T1, CBFB-MYH11, FLT3-ITD, and MLL-PTD, as well as mutations in NPM1, CEPBA, RUNX1, ASXL1, and found that no other mutation was less favorable than TP53 [8]. Treatment of AML with TP53 mutations is also troublesome, as this mutation may make AML resistant to standard forms of treatment that induce DNA damage [7]. Patients with TP53 mutations treated with anthracycline-based therapy showed response rates in only 20-30% of cases [9]. Therefore, in patients with TP53 mutations, healthcare providers should consider other treatment regimens such as decitabine, a hypomethylating agent, or venetoclax, a BCL-2 inhibitor [9]. Decitabine has demonstrated favorable response rates in TP53-associated AML, with some studies reporting up to a 66.7% response rate (n=15) [10]. Venetoclax, in combination with decitabine, has also been FDA-approved and has shown complete recovery rates in 47% of patients with TP53-associated AML (n=36) [11]. In our patient, azacitidine, a hypomethylating agent, was appropriately chosen for treatment.

In addition to a rare TP53 mutation, our patient was initially thought to have leukemia cutis. Leukemia cutis may be an early presenting factor in patients with leukemia, and thus, a high clinical suspicion for leukemia cutis must be maintained in diagnosticians. AML has been implicated in the formation of a wide variety of rashes. It is estimated that a dermatologic presentation occurs in 10-15% of patients with AML and appears commonly in patients with chromosome 8 abnormalities. When these rashes occur, they are typically violaceous, raised, non-tender papules [12]. The presentation of leukemia cutis overall is widely variable. Lesions may present on the trunk, extremities, or head. Furthermore, these lesions may be solitary, multiple, or disseminated while varying widely in color from yellowish to brown, red, or purple [13]. Morphologically the lesions can include papules, macules, plaques, nodules, ecchymoses, palpable purpura, and ulcerative lesions in all types of leukemias [14]. 

Leukemia cutis was historically diagnosed based on the clinical finding of a rash with known underlying leukemia. However, with recent advances in histopathology, the disease is now able to be better distinguished from other dermatologic disorders via biopsy [13]. Pathological results typically demonstrate tumor cells within the dermis that positively contain myeloperoxidase and cell markers such as CD15, CD43, and CD45 [15]. The presence of leukemia cutis results in a decreased overall patient survival and a decreased leukemia-specific survival [16]. More commonly, leukemias are associated with nonleukemic skin findings known as leukemids. These skin changes are nonspecific and occur in 40% of leukemias secondary to pancytopenia, drug reactions, infections, and other consequences of leukemia [17]. The nonleukemic findings in the biopsy of our patient's skin lesions indicate that he likely had a leukemoid lesion rather than leukemia cutis. Furthermore, the patient's equivocal antinuclear antibody titers may also be indicative of an underlying rheumatic process. However, due to the patient's prognosis, no further rheumatologic workup was pursued. 

In the case of our patient with AML, the presence of a rare TP53 gene mutation was indicative of a very unfavorable prognosis. Additional skin findings that were suspicious for bullous leukemia cutis raised concern for an even more dismal prognosis. It is imperative that medical professionals recognize cutaneous changes which may be evidence of underlying leukemia and obtain a biopsy of these lesions if indicated.

## Conclusions

TP53 mutations and leukemia cutis are associated with a poor prognosis in patients with AML. This report details the findings of AML associated with a rare TP53 mutation and cutaneous findings in an elderly patient who initially presented to the hospital for an acute coronary syndrome workup and was ultimately found to have pancytopenia. It is our hope that healthcare providers may use this case to become more aware of potentially fatal specifiers in AML, such as the rare TP53 mutation and the presence of cutaneous findings. Furthermore, we hope that this case reminds healthcare providers to consider underlying malignancy in the presentation of cutaneous findings consistent with leukemia cutis or a leukemoid reaction.
